# Dietary citrate supplementation enhances longevity, metabolic health, and memory performance through promoting ketogenesis

**DOI:** 10.1111/acel.13510

**Published:** 2021-10-31

**Authors:** Shou‐Zen Fan, Cheng‐Sheng Lin, Yu‐Wen Wei, Sheng‐Rong Yeh, Yi‐Hsuan Tsai, Andrew Chengyu Lee, Wei‐Sheng Lin, Pei‐Yu Wang

**Affiliations:** ^1^ Department of Anesthesiology National Taiwan University Hospital National Taiwan University Taipei Taiwan; ^2^ Graduate Institute of Brain and Mind Sciences College of Medicine National Taiwan University Taipei Taiwan; ^3^ Department of Pediatrics Taipei Veterans General Hospital Taipei Taiwan; ^4^ Neurobiology and Cognitive Science Center National Taiwan University Taipei Taiwan; ^5^ Ph.D. Program in Translational Medicine National Taiwan University and Academia Sinica Taipei Taiwan; ^6^ Taiwan International Graduate Program in Interdisciplinary Neuroscience National Taiwan University and Academia Sinica Taipei Taiwan; ^7^ Graduate Institute of Neural Regenerative Medicine College of Medical Science and Technology Taipei Medical University Taipei Taiwan

**Keywords:** dendritic spine, hippocampus, insulin, lifespan

## Abstract

Citrate is an essential substrate for energy metabolism that plays critical roles in regulating cell growth and survival. However, the action of citrate in regulating metabolism, cognition, and aging at the organismal level remains poorly understood. Here, we report that dietary supplementation with citrate significantly reduces energy status and extends lifespan in *Drosophila melanogaster*. Our genetic studies in fruit flies implicate a molecular mechanism associated with AMP‐activated protein kinase (AMPK), target of rapamycin (TOR), and ketogenesis. Mice fed a high‐fat diet that supplemented with citrate or the ketone body β‐hydroxybutyrate (βOHB) also display improved metabolic health and memory. These results suggest that dietary citrate supplementation may prove to be a useful intervention in the future treatment of age‐related dysfunction.

## Graphical abstract

Dietary citrate supplementation can induce lifespan extension in fruit flies, as well as improve memory performance and metabolic health in mice under high‐calorie diets. Our experiments demonstrate that citrate‐induced benefits require Drosophila citrate transporter Indy and act through downstream AMPK and TOR signaling to enhance ketogenesis. These results are critical in implicating citrate as an anti‐aging intervention.

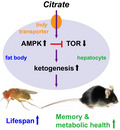

## 1 INTRODUCTION

Citrate is a pivotal substrate that mediates cellular energy metabolism. In mitochondria, citrate is produced via condensation of acetyl‐CoA and oxaloacetate by citrate synthase. It then becomes a substrate in the tricarboxylic acid (TCA) cycle and provides the major cellular ATP source. Cytosolic citrate is required for de novo fatty acid synthesis and derives from either mitochondrial release through a specific citrate carrier or extracellular import by citrate transporters across the plasma membrane. Previous studies have shown that mutations in the gene encoding the *D*. *melanogaster* and *C*. *elegans* plasma membrane citrate transporter, *I‘m Not Dead Yet* (*Indy*), result in improved metabolic fitness and lifespan extension (Rogina et al., 2000; Schwarz et al., 2015; Wang et al., 2009). A mammalian *Indy* (*mIndy*, *also called SLC13A5*) null mutation in mice also conferred superior metabolic health under high‐fat diet conditions, giving further support to the importance of citrate in metabolic regulation associated with aging (Birkenfeld et al., 2011).

Intracellular citrate can also function as a sensor for regulating energy production, since it inhibits and activates several strategic enzymes involved in glycolysis, the TCA cycle, gluconeogenesis, and fatty acid synthesis (Iacobazzi & Infantino, 2014). Because of its inhibition of glycolysis and the TCA cycle, high‐level citrate supplementation has been proposed as an anti‐cancer intervention, acting through ATP depletion to lead to arrest of cell growth and ultimately, to cell death (Lu et al., 2011; Zhang et al., 2009). A similar approach to limiting cellular energy is achieved through dietary restriction (DR, 20%–40% reduction in food intake), a well‐documented regimen that has been established as the most effective intervention for prolonging healthy lifespan across multiple species (Lin et al., 2000; Mattison et al., 2017; Tatar et al., 2014; Weindruch et al., 1986). Studies investigating the underlying mechanism of DR have identified signaling pathways involving nutrient‐sensing and metabolism, such as sirtuins (Sir), AMP‐activated protein kinase (AMPK), target of rapamycin (TOR), and downstream ketogenesis pathways (Kaeberlein et al., 2005; Lin et al., 2000; Newman et al., 2017; Roberts et al., 2017; Stenesen et al., 2013; Teng et al., 2019). Interventions acting on these targets often result in improved metabolic health and lifespan extension (Madeo et al., 2019).

The influence of chronic citrate treatment on metabolism, cognition, and aging at the organismal level remains unknown. Using fruit flies and mice as model organisms, we show that dietary citrate supplementation can promote longevity, improve metabolic health, and enhance memory performance, through a mechanism associated with the AMPK, TOR, and ketogenic pathways. Our findings thus have critical implications for use of citrate in the treatment of diseases associated with aging.

## 2 RESULTS

### 2.1 Citrate supplementation extends lifespan

**FIGURE 1:**
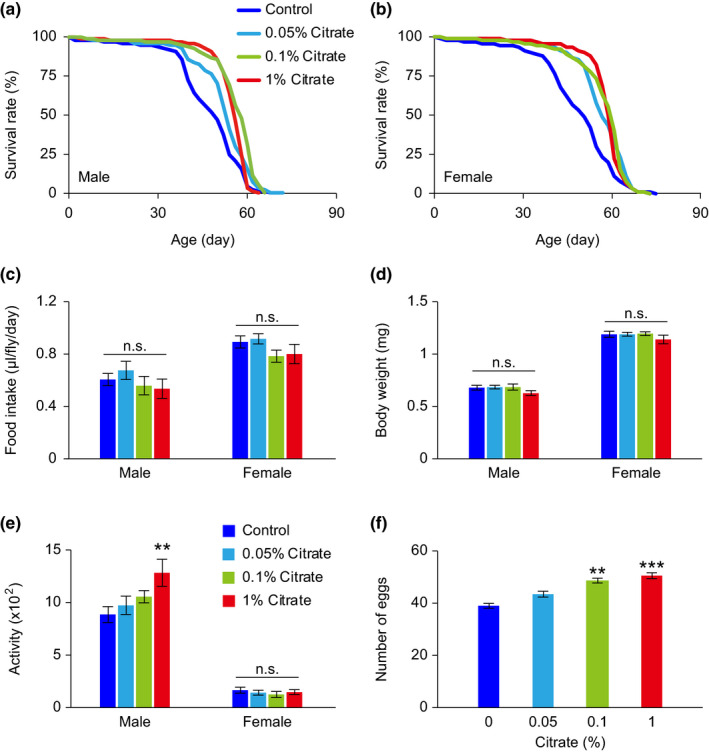
Physiological status of *w^1118^ Drosophila melanogaster* treated with various concentrations of citrate. (a, b) Lifespans of male (a) and female (b) *w^1118^* flies treated with different concentrations of citrate. (c–f) Food intake (c), body weight (d), locomotor activity (e), and fecundity (f) of flies treated with different concentrations of citrate for 10 days. Detailed statistical analyses for the lifespans are shown in Table S2. The other data are expressed as mean ± SEM (*n* = 10–42 vials, 10 flies per vial). Not significant (n.s.), **p < 0.01, ***p < 0.001, compared to the control group by one‐way ANOVA with Fisher's LSD post hoc test

To explore the action of dietary citrate supplementation on aging, we began by examining the effect of citrate on lifespan regulation in wild‐type *w^1118^* flies. We performed our *Drosophila* lifespan experiments in 1‐liter population cages with a relatively high‐calorie diet (15% yeast and 5% sucrose). Flies on this regimen display a shorter lifespan, heavier body weight, reduced physical activity, and increased fecundity, compared to flies fed lower calorie diets (Lin et al., 2014; Lin et al., 2018). We found that addition of 0.01%, 0.1%, or 1% citrate to the diet significantly induced lifespan extension in both male and female *w^1118^* flies, with a plateau for lifespan extension reached at 0.1% citrate supplementation (Figure 1a,b). Citrate supplementation did not significantly affect food intake or body weight of flies, although there was a small trend toward reduced food intake and body weight in both sexes of flies treated with the higher concentrations of citrate (Figure 1c,d). Moreover, treatment with higher concentrations of citrate also notably improved spontaneous locomotor activity and fecundity in male and female flies, respectively (Figure 1e,f). These data suggest that citrate supplementation can promote longevity and some aspects of organismal fitness in fruit flies.

### 2.2 Citrate‐induced altered energy metabolism

**FIGURE 2:**
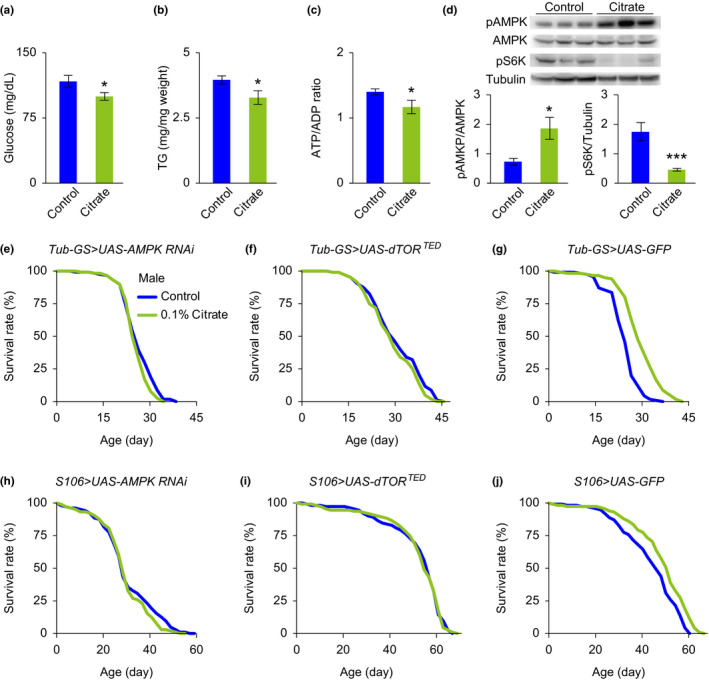
Citrate‐induced lifespan extension is associated with the AMPK and TOR pathways. (a–c) The levels of hemolymph glucose (a), total triglyceride (b), and ATP/ADP ratio (c) of male *w^1118^* flies treated with vehicle or 0.1% citrate for 10 days. (d) Representative Western blots and quantitative analyses showing that phosphorylated AMPK and phosphorylated S6K are upregulated and downregulated, respectively, in male *w^1118^* flies treated with 0.1% citrate for 10 days, compared with vehicle‐treated controls. (e, f) Lifespans of male mutant flies with RU486‐induced systemic inhibition of AMPK (e, *Tub*‐*GS*>*UAS*‐*AMPK RNAi*) and dTOR (f, *Tub*‐*GS*>*UAS*‐*dTOR^TED^*), treated with vehicle or 0.1% citrate. (h, i) Lifespans of male mutant flies with RU486‐induced fat body‐specific inhibition of AMPK (h, *S106*>*UAS*‐*AMPK RNAi*) and dTOR (i, *S106*>*UAS*‐*dTOR^TED^*), treated with vehicle or 0.1% citrate. (g, j) Lifespans of male mutant flies with RU486‐induced systemic (g, *Tub*‐*GS*>*UAS*‐*GFP*) and fat body‐specific (j, *S106*>*UAS*‐*GFP*) overexpression of GFP, treated with vehicle or 0.1% citrate. Detailed statistical analyses for the lifespans are shown in Table S2. The other data are expressed as mean ± SEM (*n* = 6–9 samples). *p < 0.05, ***p < 0.001 compared to the control group by Student's *t* test

Since citrate is known to suppress glycolysis and the TCA cycle, we hypothesized that excessive citrate intake might negatively impact intracellular energy homeostasis. To test this hypothesis, we measured hemolymph glucose and total triglyceride levels, as well as the ATP/ADP ratio, as a representation of the energy status of flies. We found that 0.1% citrate supplementation for 10 days induced lower levels for all these measurements in *w^1118^* flies compared to the vehicle groups, indicating reduced energy status in citrate‐treated flies (Figure 2a–c). In order to confirm these observations, we also performed Western blots to measure the protein phosphorylation of AMPK and S6 Kinase in flies, as phosphorylation levels of these proteins reflect the relative activity of AMPK and TOR in the energy‐sensing and metabolic pathways (Grabacka et al., 2016). We found that 10 days of citrate supplementation induced increased AMPK activity, but reduced TOR activity in flies, further supporting our observations of reduced energy status in citrate‐treated flies (Figure 2d). These results are consistent with previous reports that AMPK is activated in response to a lower energy level and that it blocks anabolic processes by antagonizing TOR signaling (Grabacka et al., 2016).

If the AMPK and TOR pathways are critical in mediating citrate‐induced lifespan extension, we would expect to no longer see a citrate‐induced lifespan extension when these two pathways are genetically blocked. We employed the RU486 (mifepristone)‐inducible UAS‐Gal4 system to overexpress AMPK RNAi and a dominant negative transgene of *Drosophila* TOR (dTOR^TED^), in order to genetically inhibit these two pathways specifically in the adult stage of flies. Our data show that citrate did not induce a lifespan extension in flies with systemic overexpression of UAS‐AMPK RNAi or UAS‐dTOR^TED^, using a tubulin gene‐switch (Tub‐GS) Gal4 driver (Figures 2e,f, and S1a,b). Since we also observed that the *Drosophila* citrate transporter *Indy* is required for citrate supplementation induced lifespan extension in our experiments (Figure S2a,b), and the *Indy* gene is highly expressed in the fat body of flies (Rogina et al., 2000), we further examined whether functional signaling of AMPK and TOR in the fat body is essential for citrate‐induced lifespan extension. Our data show that fat body‐specific (S106 Gal4) inhibition of AMPK and TOR abrogated citrate‐induced lifespan extension (Figures 2h,I and S1d,e). These negative observations are valid because citrate induces significant lifespan extension in our control flies in the RU486‐inducible UAS‐Gal4 system, including flies overexpressing green fluorescent protein (GFP) (Figures 2g,j and S1c,f), and flies of different genetic backgrounds in the absence of the RU486 inducer (Figure S3a–p). Moreover, citrate supplementation does not augment the lifespan extension of flies with fat body‐specific AMPK overexpression, further supporting our hypothesis that the AMPK pathway mediates citrate‐induced lifespan extension (Figure S4a,b).

### 2.3 Ketogenesis mediates citrate‐induced lifespan extension

**FIGURE 3:**
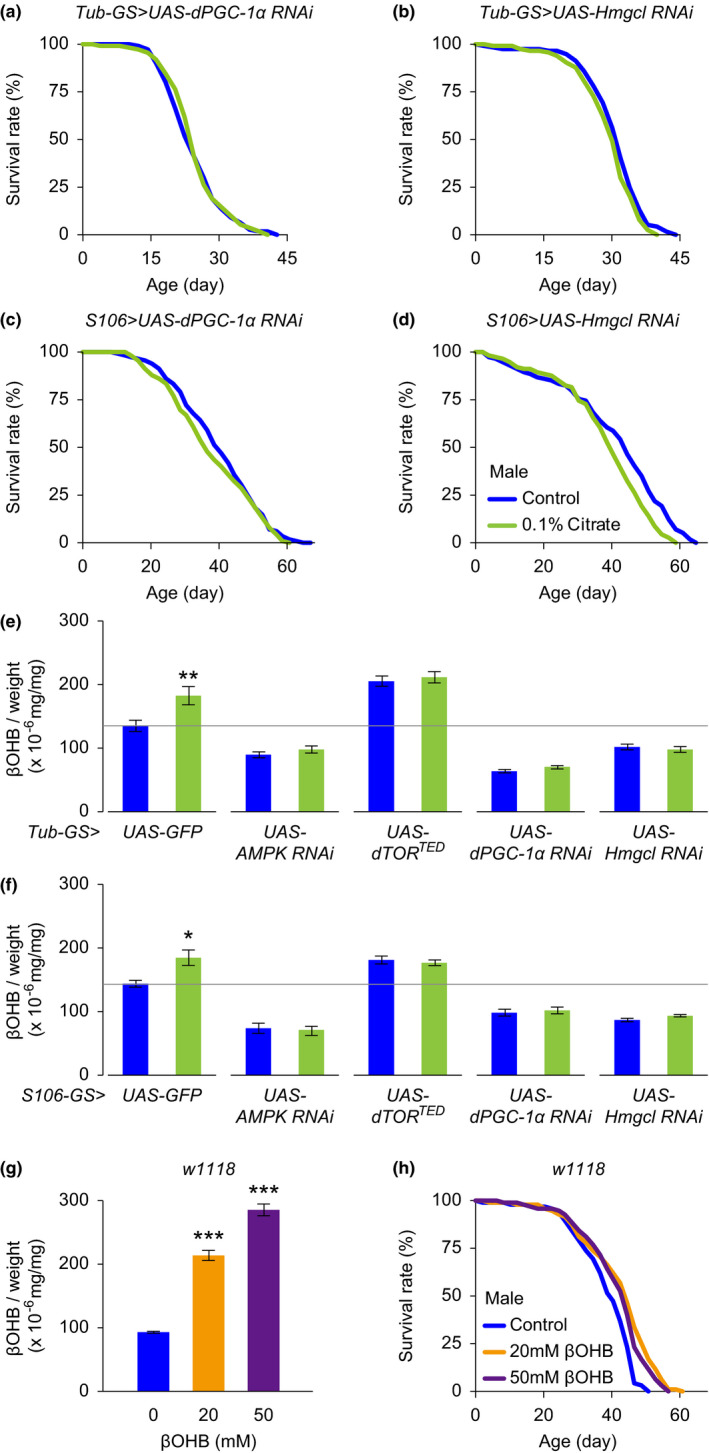
Ketogenesis mediates citrate‐induced lifespan extension. (a, b) Lifespans of male mutant flies with RU486‐induced systemic inhibition of PGC‐1α (a, *Tub*‐*GS*>*UAS*‐*PGC*‐*1α RNAi*) and Hmgcl (b, *Tub*‐*GS*>*UAS*‐*Hmgcl RNAi*), treated with vehicle or 0.1% citrate. (c, d) Lifespans of male mutant flies with RU486‐induced fat body‐specific inhibition of PGC‐1α (c, *S106*>*UAS*‐*PGC*‐*1α RNAi*) and Hmgcl (d, *S106*>*UAS*‐*Hmgcl RNAi*), treated with vehicle or 0.1% citrate. (e, f) Citrate treatment for 10 days increases βOHB levels in male genetic control flies (*Tub*‐*GS*/*S106*>*UAS*‐*GFP*), but not in male mutant flies with systemic and fat body‐specific overexpression of AMPK RNAi (*Tub*‐*GS*/*S106*>*UAS*‐*AMPK RNAi*), dTOR^TED^ (*Tub*‐*GS*/*S106*>*UAS*‐*dTOR^TED^*), PGC‐1α RNAi (*Tub*‐*GS*/*S106*>*UAS*‐*PGC*‐*1α RNAi*), and HMGL RNAi (*Tub*‐*GS*/*S106*>*UAS*‐*Hmgcl RNAi*). (g) Dietary βOHB supplementation for 10 days dose‐dependently increases βOHB in male flies. (h) Lifespans of male *w^1118^* flies treated with various concentrations of βOHB. Detailed statistical analyses for the lifespans are shown in Table S2 and S3. The other data are expressed as mean ± SEM (*n* = 9–10 samples). *p < 0.05, **p < 0.01, ***p < 0.001 compared to the control group by Student's *t* test or one‐way ANOVA with Fisher's LSD post hoc test

Increased ketogenesis is a prominent feature of prolonged fasting as well as of chronic energy stress. The metabolic switch from carbohydrates to fatty acids and ketones when experiencing energy shortage is tightly regulated at multiple steps, involving downstream signaling of AMPK and TOR (Carling et al., 1989; Sengupta et al., 2010). We next genetically disrupted the ketogenetic pathway by overexpressing RNAi for peroxisome proliferator‐activated receptor gamma coactivator 1‐alpha (PGC‐1α) and hydroxymethylglutaryl‐CoA‐lyase (HMGCL), which act downstream of AMPK and mTOR, respectively, in regulating fatty acid oxidation and ketogenesis. We found that both systemic and fat body‐specific inhibition of PGC‐1α and HMGCL in flies eliminated the citrate‐induced lifespan extension (Figures 3a–d and S5a–d).

Consistent with these observations, we also found that 10 days of citrate supplementation significantly increased the level of β‐hydroxybutyrate (βOHB), one of the three major ketone bodies, in our genetic control flies (*Tub*‐*GS*>*UAS*‐*GFP* and *S106*>*UAS*‐*GFP*), but not in mutant flies with systemic and fat body‐specific overexpression of AMPK RNAi, dTOR^TED^, PGC‐1α RNAi and HMGL RNAi (Figures 3e,f and S5e,f). It is of particular interest that inhibition of AMPK, PGC‐1α, and HMGCL repressed ketogenesis, while disruption of TOR signaling enhanced it, providing further evidence of an antagonistic interaction between AMPK and TOR signaling.

In order to test our hypothesis that ketone bodies exerted the lifespan‐extending activity in our experimental setups, we treated flies with various concentrations of βOHB, adding it to the high calorie fly food. We found that βOHB intake increased the level of βOHB dose‐dependently, with significant lifespan extension observed in both male and female flies (Figures 3g,h and S5g,h).

### 2.4 *Citrate administration improves metabolic health in mice fed on a high*‐*fat diet*

**FIGURE 4:**
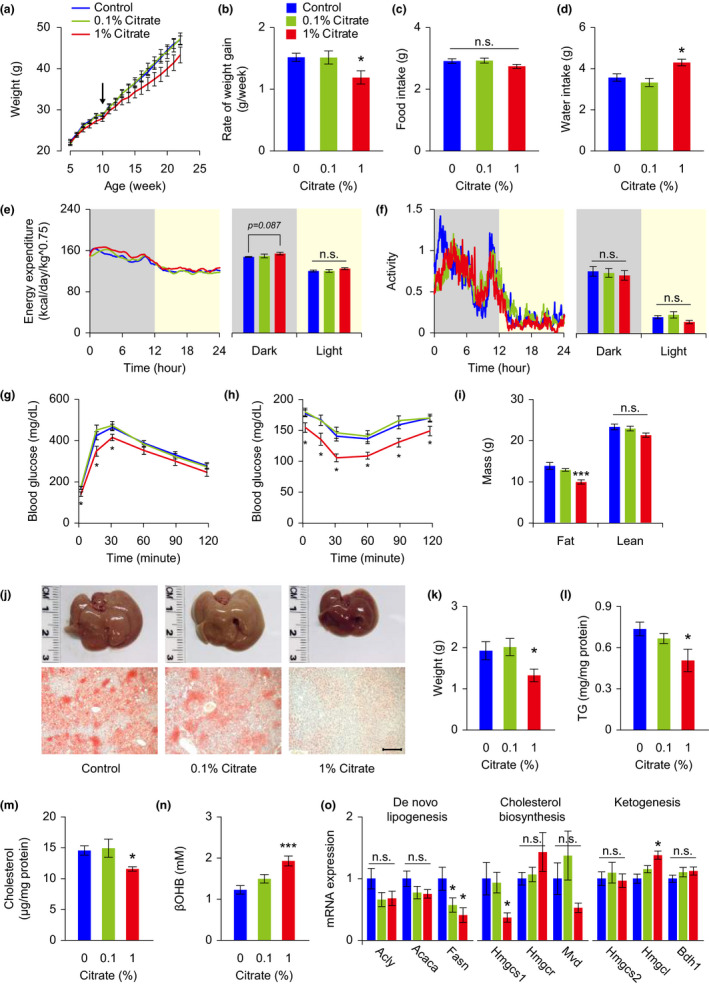
Citrate administration improves metabolic health in mice fed on a high‐fat diet. Mice fed on a high‐fat diet were treated with various concentrations of citrate from 10 weeks of age (arrow). Metabolic health of mice was examined between 20 and 28 weeks of age. (a–d) Body weight (a), rate of weight gain (b), food intake (c), and water consumption (d) were monitored and analyzed. (e, f) Energy expenditure (e), and spontaneous locomotor activity (f) were analyzed in mice receiving vehicle and citrate treatments. (g, h) Glucose tolerance test (g) and insulin tolerance test (h) were conducted in mice receiving vehicle and citrate treatments. (i) Body composition was analyzed in mice receiving vehicle and citrate treatments. (j–m) Mice receiving 1% citrate treatment showed reduced liver weight (j, k) and decreased hepatic lipid accumulation, including reduced oil red O staining (j, lower panels) and lower levels of triglyceride (TG, l) and cholesterol (m). (n) Citrate administration dose‐dependently enhanced βOHB levels in the blood. (o) mRNA measurements of genes associated with de novo lipogenesis, cholesterol biosynthesis, and ketogenesis, from livers of mice receiving vehicle and citrate treatments. ATP citrate lyase (Acly), acetyl‐CoA carboxylase (Acaca), fatty acid synthase (Fasn), HMG‐CoA synthetase 1 (Hmgcs1), HMG‐CoA reductase (Hmgr), mevalonate diphosphate decarboxylase (Mvd), HMG‐CoA synthetase 2 (Hmgcs2), HMG‐CoA lyase (Hmgcl), β‐hydroxybutyrate dehydrogenase 1 (Bdh1). Data are expressed as mean ± SEM (*n* = 8 mice for each group in the whole‐body metabolic analyses, *n* = 10 mice for each group in all the other tests). Not significant (n.s.), *p < 0.05, **p < 0.01, ***p < 0.001 compared to the control group by one‐way ANOVA with Fisher's LSD post hoc test. Scale bar = 200 μm

Our fly studies provide strong evidence that the anti‐aging effects of citrate under high‐calorie diet conditions are likely to act through promoting ketogenesis. To further evaluate the translational potential of citrate supplementation in higher order organisms, we tested the effect of citrate administration in mice fed a high‐fat diet. Mice that received 1% citrate in the drinking water showed significantly reduced weight gain, but those receiving 0.1% did not (Figure 4a,b). Although food intake was not affected, elevated concentration of citrate in the drinking water notably increased water consumption of the mice (Figure 4c,d). This phenomenon could be due to a higher salt concentration in the drinking water, deriving from use of sodium hydroxide as an acid‐neutralizing agent for the citric acid.

Mice treated with 1% citrate showed a trend toward higher oxygen consumption and CO_2_ production, and thus toward slightly higher energy expenditure, in the dark phase of the light‐dark cycle, which could contribute to the lower body weight gain observed (Figures 4a,b,e and S6a,b). There were no differences observed in the respiratory quotient or spontaneous locomotor activity of mice in the home cage (Figure 4f and S6c). Mice treated with 1% citrate also exhibited better glucose metabolism when tested in the glucose tolerance and insulin tolerance tests (Figure 4g,h).

Mice receiving 1% citrate showed altered body composition, with significantly reduced fat mass, but unchanged lean mass (Figure 4i). The reduced fat content is largely attributable to lower lipid accumulation in the liver, since lower liver weight and hepatic lipid levels, such as triglycerides and cholesterol, were recorded in these mice (Figure 4j–m). There was a small trend toward reduced adiposity in the white adipose tissues of citrate‐treated mice, compared to controls (Figure S7a,b). Increased circulating βOHB was detected in a dose‐dependent manner in citrate‐treated mice under fasting conditions, which is in agreement with our observations in citrate‐treated flies (Figure 4n). Pursuing this line of thought, we discovered reduced mRNA expression of some genes involved in *de novo* lipogenesis and cholesterol biosynthesis, but increased mRNA expression in genes involved in ketogenesis regulation when we examined gene expression patterns in liver tissue from citrate‐treated mice (Figure 4o).

### 2.5 Citrate administration improves memory performance of mice fed on a high‐fat diet

**FIGURE 5:**
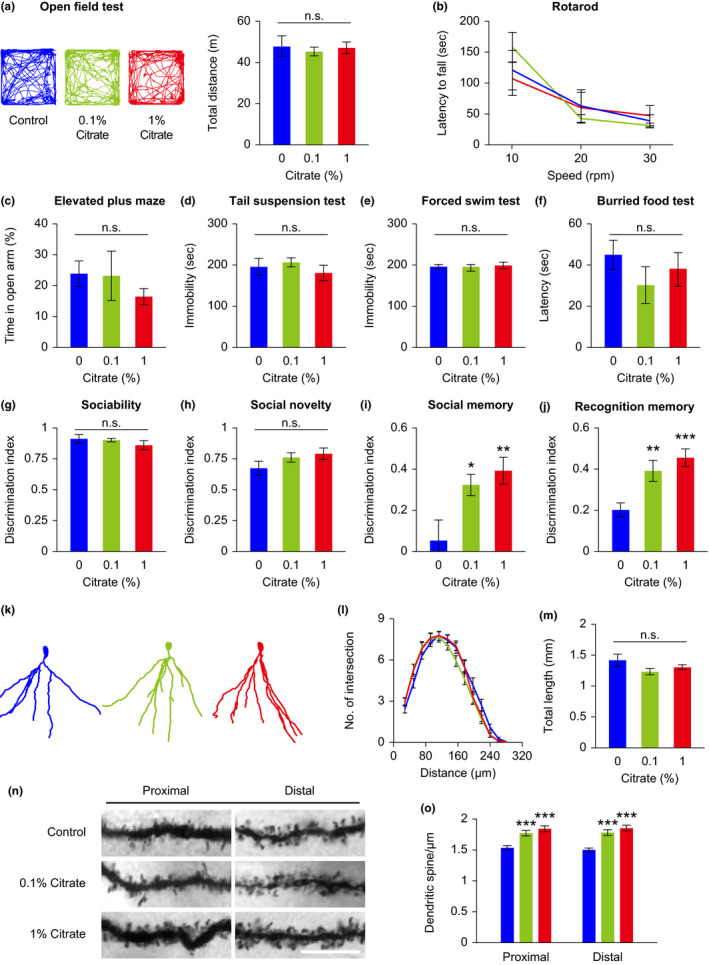
Citrate administration affects mouse cognitive function. Mice fed on a high‐fat diet were treated with different concentrations of citrate from 10 weeks of age. Behavioral tests of mice were carried out between 16 and 20 weeks of age. (a–j) Mice were subjected to the open field test (a, representative moving path and total travel distance), rotarod test (b), elevated plus maze test (c), tail suspension test (d), forced swim test (e), buried food test (f), 3‐chamber social test (g–i), and novel object recognition test (j). (k–o) Structural analyses of hippocampal DG granule cells from vehicle‐ and citrate‐treated mice were analyzed at 28 weeks of age. DG granule cells were reconstructed (k), and the dendritic profile (l) and dendritic length (m) were analyzed. Representative micrographs of proximal (<50 μm from the soma) and distal (>150 μm from the soma) dendrites (n), and quantitative spine density (o) of DG granule cells. Data are expressed as mean ± SEM (*n* = 10 mice for behavioral tests, *n* = 29–30 neurons, and 58–60 dendritic segments for structural analyses). Not significant (n.s.), *p < 0.05, **p < 0.01, ***p < 0.001 compared to the control group by one‐way ANOVA with Fisher's LSD post hoc test. Scale bar = 10 μm

Because metabolic status is often linked to cognitive health, we employed a pool of behavioral tasks to evaluate the cognitive function of citrate‐treated mice. Mice treated with 0.1% and 1% citrate exhibited normal spontaneous locomotor activity and general locomotor coordination in the open field and rotarod tests (Figure 5a,b). Mice receiving 0.1% and 1% citrate supplementation showed no differences from controls in anxiety‐ or depression‐like behavior, as measured in the elevated plus maze, tail suspension, and forced swim tests (Figure 5c–e). Citrate‐treated mice displayed normal sociability and social novelty behaviors, but surprisingly, both 0.1% and 1% citrate treatments induced superior social recognition memory compared to that of control mice in the three‐chamber test (Figure 5g–i). The improved performance in social recognition memory could not be attributed to an abnormality in olfactory function, important in murine social interaction, since these mice behaved normally in the buried food test (Figure 5f). However, we used the novel object recognition test to substantiate the improved recognition memory in these citrate‐treated mice (Figure 5j).

Morphological changes in hippocampal neurons have been shown to couple with altered memory performance in animals (Teng et al., 2019). We performed the Golgi‐Cox impregnation staining method, and reconstructed dendritic profiles of dentate gyrus (DG) granule cells in the mouse hippocampus, using Neurolucida software. We found that both 0.1% and 1% citrate treatment in the context of the high‐calorie diet induced increased dendritic spine density without affecting dendritic complexity or dendritic length (Figure 5k–o).

### 2.6 Ketone body supplementation mimics citrate‐induced metabolic health and memory improvement

**FIGURE 6:**
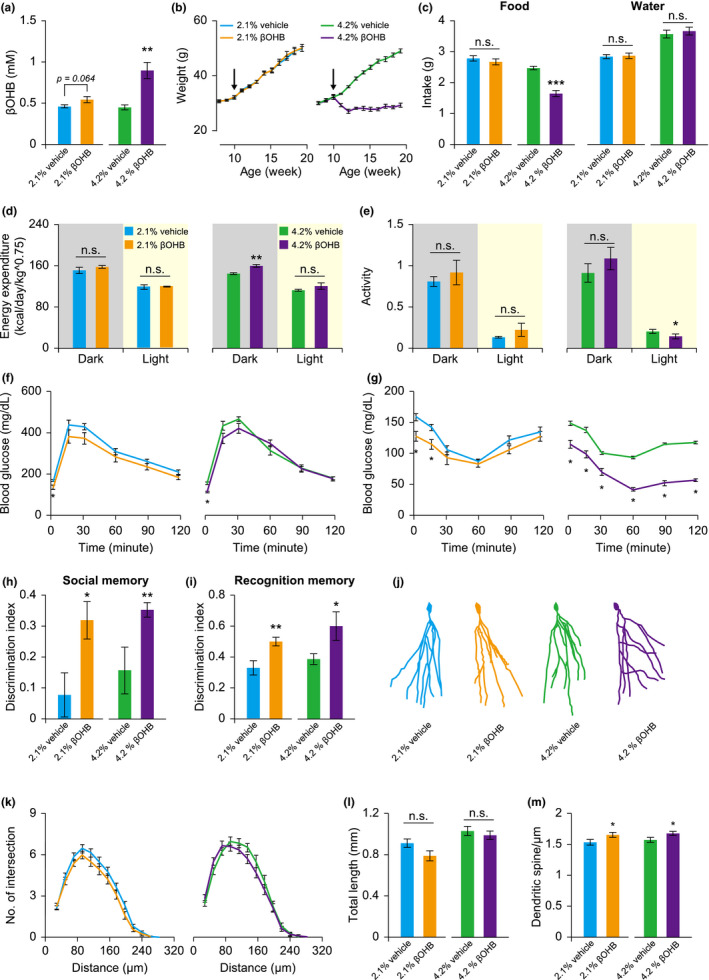
βOHB administration improves metabolic health and memory performance of mice. Mice fed on a high‐fat diet were treated with different concentrations of βOHB and/or vehicle from 10 weeks of age (arrow). Metabolic health and behavioral tests were carried out between 16 and 24 weeks of age. (a–c) Circulating βOHB (a), body weight (b), and food and water intake (c) were monitored and analyzed. (d, e) Energy expenditure (d), and spontaneous locomotor activity (e) were analyzed in mice receiving vehicle and βOHB treatments. (f, g) Glucose tolerance test (f) and insulin tolerance test (g) were conducted in mice receiving vehicle and βOHB treatments. (h, i) Memory performance of mice was evaluated using a 3‐chamber social test (h) and novel object recognition test (i), respectively. (j–m) DG granule cells were reconstructed (j), and the dendritic profile (k), dendritic length (l), and spine density of distal dendrites (m) were analyzed. Data are expressed as mean ± SEM (*n* = 4–5 for whole‐body metabolic analyses, *n* = 8–10 mice for behavioral and the other tests, *n* = 26–39 neurons, and 30–31 dendritic segments for structural analyses). Not significant (n.s.), *p < 0.05, **p < 0.01, ***p < 0.001 compared to the control group by Student's *t* test

Our experiments have demonstrated that citrate administration can significantly improve metabolic health and memory performance, and suggest that increased ketogenesis could play a critical role in mediating these beneficial effects. We confirmed this possibility in our experimental model by treating mice with βOHB sodium/potassium or vehicle control in drinking water. We found that mice receiving 2.1% and 4.2% βOHB sodium/potassium had increased circulating levels of βOHB compared to their vehicle (sodium/potassium salt mixture) controls (Figure 6a). Mice receiving 2.1% βOHB sodium/potassium showed normal body weight gain, food intake, and water consumption (Figure 6b,c). However, mice receiving 4.2% βOHB sodium/potassium displayed reduced body weight, accompanied by reduced food intake (Figure 6b,c). Although the βOHB sodium/potassium treatment did not alter water consumption of the mice, higher salt concentration significantly increased water ingestion, compared to lower salt groups (Figure 6c).

In whole‐body metabolic analyses, mice receiving 2.1% βOHB sodium/potassium showed normal oxygen consumption, CO_2_ production, energy expenditure, respiratory quotient, and spontaneous locomotor activity in the home cage (Figures 6d,e, and S6d–f). On the other hand, mice receiving 4.2% βOHB sodium/potassium showed increased levels of all these metabolic indexes in the dark phase, but without changes in respiratory quotient or spontaneous locomotor activity (Figures 6d,e, and S6g–i). Thus, reduced food intake and higher energy expenditure may contribute to the decreased body weight observed in the 4.2% βOHB sodium/potassium‐treated mice. We also monitored glucose metabolism in these mice and found that both 2.1% and 4.2% βOHB sodium/potassium treatments induced a lower level of fasting blood glucose (Figure 6f,g). Although βOHB sodium/potassium only slightly improved glucose metabolism as measured by the glucose tolerance test, it significantly enhanced insulin sensitivity in the insulin tolerance test (Figure 6f,g).

Similar to the mice receiving citrate supplementation, those receiving 2.1% and 4.2% βOHB sodium/potassium treatment showed enhanced social and object recognition memory, accompanied by an increase in dendritic spine density of the hippocampal DG granule cells (Figure 6h,i,m). Neuronal complexity and total dendritic length were largely normal (Figure 6j–l). Mice receiving 4.2% βOHB sodium/potassium also displayed higher spontaneous locomotor activity and motor coordination in the open field and rotarod tests, suggesting improved motor fitness in these mice (Figure S8a–c). There were no obvious changes in other behavioral tasks, including anxiety‐like behavior, depression‐like behavior, olfactory function, or social behavior (Figure S8d–i).

## 3 DISCUSSION

Identification of pro‐longevity pathways and translation of these findings to therapeutic strategies have the potential to benefit human well‐being through decreased healthcare costs and diminished need for healthcare access. With a view to these realities, our current studies have identified citrate as an effective intervention with potential for extending lifespan, improving metabolic health, and enhancing memory performance in experimental animals under high‐calorie diet conditions. Genetic manipulation and pharmacological treatment of both fruit flies and mice in our studies point to a molecular mechanism underlying these beneficial effects that is associated with the AMPK, TOR, and ketogenetic pathways.

Since lifespan experiments in mice are time‐consuming, we therefore take advantage of the shorter life cycle of *Drosophila* to report the anti‐aging activity of dietary citrate supplementation. The *Drosophila* lifespan assay is well‐established and widely accepted in the field of aging research (Linford et al., 2013). Although we currently have no evidence that citrate supplementation can induce lifespan extension in mice, the improved metabolic health and cognitive function observed in citrate‐treated mice are often linked to pro‐longevity interventions in animal studies. For example, DR has been shown to improve metabolic health or cognitive performance in both young and aged animals (Dommerholt et al., 2018; Sohal et al., 1994; Teng et al., 2019). The DR mimetic, rapamycin (TOR inhibitor), has been shown not only to induce lifespan extension, but also, age‐independently, to improve cognitive performance and metabolic health in mice (Neff et al., 2013). Accordingly, our results indicate that citrate supplementation can have important implications for improving both healthspan and lifespan of model organisms.

Previous studies have examined the effects of TCA cycle metabolite supplementation on lifespan regulation in *C*. *elegans* (Chin et al., 2014; Edwards et al., 2015). However, results have been variable and sometimes contradictory. For example, Chin et al. (2014) reported that 8mM α‐ketoglutarate and succinate, but not citrate or isocitrate, supplementation induced a 25%–50% lifespan extension in *C*. *elegans*, while Edwards et al. (2015) showed that 1–10 mM α‐ketoglutarate, succinate, and isocitrate, but not citrate, induced a 3%–15% lifespan extension in *C*. *elegans*. These discrepancies may reflect different culture conditions for the worms used in these two separate studies. Nevertheless, neither study found a citrate‐induced lifespan extension for *C*. *elegans* under the particular experimental conditions. We speculate that species and dietary differences between the worm and fly studies may be the main issues requiring further investigation. Although a previous study reported that citrate supplementation can improve healthspan of flies, including reduced body weight, improved negative geotaxis behavior, and protection from oxidative stress, the pro‐longevity effects of citrate were not examined (Panchala et al., 2016). It is worth mention that the citrate concentrations used in this particular study were 150 mM and 300 mM, which are about 50–100 times higher than those used in our current study (0.1% ≈ 5.2 mM) and in most published work involving TCA cycle metabolites (1–10 mM) in lifespan regulation (Chin et al., 2014; Edwards et al., 2015; Williams et al., 2009).

Studies of the *C*. *elegans*, *D*. *melanogaster*, and mammalian plasma membrane citrate transporter, *Indy* (*SLC13A5* for mammalian) have demonstrated a critical role for citrate in regulating metabolic homeostasis and lifespan extension at the organismal level (Birkenfeld et al., 2011; Rogina et al., 2000; Schwarz et al., 2015; Wang et al., 2009). While these studies suggest that deficiency in cellular citrate transport can induce a metabolic status akin to DR, our data show that excess citrate supplementation can also result in low energy status, increased longevity, and improved metabolic health in flies and mice. Accordingly, we postulate that citrate is an intracellular energy sensor that regulates metabolic homeostasis in a biphasic manner. *SLC13A3* is another mammalian homolog of the *Indy* gene, and it exhibits more than 10‐fold greater transport efficacy for succinate compared to citrate (Chen et al., 1999). Although physiological outcomes of *SLC13A3* genetic deficiency have not been reported, overexpression of *SLC13A3* in human diploid cells and in primary renal tubular cells has been linked to premature cellular senescence through induction of oxidative stress (Ma et al., 2016). Whether *SLC13A3* overexpression‐induced cellular senescence is associated with excessive intracellular citrate accumulation remains to be determined.

Our genetic experiments in flies reveal an important role for ketogenesis in mediating citrate‐induced lifespan extension. Dietary βOHB administration in our fly experimental model consistently resulted in an increased lifespan, similar to what was observed in *C*. *elegans* (Edwards et al., 2014). Another striking finding involving ketone body‐associated longevity regulation is the lifespan‐extending effect of ketogenic diets demonstrated in recent mouse studies (Newman et al., 2017; Roberts et al., 2017). Our results in mice detail the health benefits of βOHB supplementation, similar to findings of previous reports, including body weight loss, increased energy expenditure, and improved glucose hemostasis, but with a significantly reduced food intake (Kashiwaya et al., 2010; Srivastava et al., 2012). Although the inhibitory effect of βOHB on food intake is well‐documented, the underlying cellular and molecular mechanisms are not fully understood. A previous study suggests that local production of βOHB by astrocytes is essential for βOHB‐induced appetite suppression, through its action on fatty acid‐sensing neurons in the ventromedial hypothalamus (Le Foll et al., 2014). This raised the possibility that βOHB as a dietary supplement may act directly on neurons to suppress feeding, while supplementary citrate may require further metabolic steps to initiate ketogenesis. This discrepancy might explain why dietary supplementation of citrate did not induce an obvious feeding suppression such as that seen in mice receiving supplementary βOHB. Lower body weight in βOHB‐treated mice also contributes to improved motor learning and motor control, which are commonly seen in animals under DR (Ingram et al., 1987).

In addition to their role as an alternative energy resource during carbohydrate deprivation, some evidence suggests that ketone bodies may act as signaling molecules. βOHB has been shown to bind and inhibit the activity of class I histone deacetylases (Riggs et al., 1977; Shimazu et al., 2013), possibly accounting for the observation of histone hyperacetylation in cells treated with βOHB both *in vitro* and *in vivo*, and βOHB treatment also leads to transcriptional changes in genes associated with cell survival and oxidative stress responses (Shimazu et al., 2013). Two recent studies invoked a similar mechanism in showing that βOHB‐induced expression of brain‐derived neurotrophic factor (BDNF) in cortical and hippocampal neurons, both in cell culture and in mice (Marosi et al., 2016; Sleiman et al., 2016). Further, βOHB‐induced BDNF expression could play an important role in regulation of the synaptic plasticity that contributes to increased spine formation and improved memory performance observed in this study. Our recent work and a previous report point out that deletion of the mammalian citrate transporter *Indy* in mice can induce improved metabolic health, memory performance, and increased ketogenesis (Birkenfeld et al., 2011; Fan et al., 2021). These observations further support our hypothesis of a biphasic effect of citrate in the regulation of physiology. Interestingly, both citrate supplementation and mammalian *Indy* knockout mice display improved performance in memory tests, but not in the other tests of cognitive function examined in this study. Higher expression levels of mammalian *Indy* in the cerebral cortex and hippocampus could be a possible explanation for this discrepancy, though the expression pattern of the other citrate transporters in the brain has not been fully investigated (Fan et al., 2021). Further experiments will be helpful in dissecting the interaction of citrate supplementation and the various citrate transporters in cognitive regulation.

In summary, our study identifies citrate as a promising anti‐aging supplement with potential for improving multiple aspects of health and aging in fruit flies and in mice. We propose that a molecular mechanism associated with AMPK, TOR, and ketogenesis mediates these beneficial effects. By shedding light on this mechanism, our results may pave the way for future development of novel interventions for delaying aging and treating age‐related dysfunction.

## 4 EXPERIMENTAL PROCEDURES

### 4.1 Flies and dietary manipulation

*w^1118^*, *Indy 206*, *tubulin*‐*gene switch (GS) Gal4*, *S106*‐*GS Gal4*, *UAS*‐*GFP*, *UAS*‐*mCherry*‐*AMPK*, *UAS*‐*AMPK RNAi*, *UAS*‐*dTor^TED^*, *UAS*‐*PGC*‐*1α RNAi*, and *UAS*‐*Hmgcl RNAi* flies were maintained on food containing 5% sucrose, 5% yeast, 5% cornmeal food, 1% agar, and 0.23% Tegosept (Apex Bioresearch Products). The yeast concentration was adjusted to 15% for the lifespan experiments. All flies were backcrossed into the *w^1118^* background for at least 5 generations and maintained in a humidified, temperature‐controlled incubator with 12‐h on/off light cycle at 25°C throughout the experiment. Various concentrations of citrate, βOHB sodium salt (Sigma), and 50mM NaCl (vehicle control for βOHB sodium salt) were added to the fly food. For the gene switch experiment, 200 μM RU486 or vehicle (0.5% ethanol) was added to the fly food.

### 4.2 Lifespan assays

Lifespan assays were performed as described previously, with minor modifications (Huang et al., 2015; Lin et al., 2014; Wang et al., 2009). Briefly, newly eclosed flies (approximately 110 males and 110 females) were collected and introduced to each 1 L population cage. Fresh food was provided every other day, and the number and sex of dead flies were counted. Each lifespan assay was conducted in at least two independent cages, and data were merged for the statistical analyses.

### 4.3 Food intake, body weight, locomotor activity, and fecundity measurements

We performed the CAFE assay (Ja et al., 2007) with minor modifications to measure the food intake of flies. Briefly, ten 10‐day‐old male or female flies were transferred to fresh vials that had four 5uL capillary tubes loaded with the liquid extract of autoclaved fly food (without agar). Fresh capillary tubes were provided daily, and the food intake of flies was monitored for 5 days. The body weight of flies was measured for ten 10‐day‐old anesthetized male or female flies, using a microbalance (Sartorious). For determining spontaneous locomotor activity, ten 10‐day‐old male or female flies were placed in standard chambers equipped with circular rings of infrared light sources at various heights, in which any beam breaks are recorded as activity (TriKinetics Inc.). Female fecundity was measured by counting the number of eggs laid in 24 h by 10‐day‐old female flies.

### 4.4 Western blot analysis

Flies were lysed in radioimmunoprecipitation assay buffer (Thermo Fisher Scientific) and proteins were then separated by SDS‐PAGE using standard procedures (Wang et al., 2005). The antibodies used were mouse anti‐AMPK (Abcam), rabbit anti‐phospho‐AMPK (Cell Signaling), rabbit anti‐phospho‐*Drosophila* p70 S6 Kinase (Cell Signaling), and mouse anti‐α Tubulin (GeneTex). Protein signals were detected with horseradish peroxidase‐conjugated secondary antibodies and ECL reagent (Thermo Fisher Scientific). Immunoblots were quantified using Image J software.

### 4.5 Animal and food manipulations

All experimental protocols followed local animal ethics regulations and were approved by National Taiwan University College of Medicine and College of Public Health Institutional Animal Care and Use Committee. Male C57BL/6 mice were obtained from the Laboratory Animal Center, National Taiwan University College of Medicine. All mice were group‐housed (2–5 mice per cage) and maintained in an animal room at a controlled temperature of 22–24℃ and 50%–55% humidity, under a 12‐h light/dark cycle. Various concentrations of citrate (Sigma) were dissolved in drinking water with pH adjusted to between 7.3 and 7.4 by addition of sodium hydroxide (Sigma). βOHB sodium/potassium (KetoForce) was diluted with drinking water to have a final βHB concentration of 4.2% or 2.1% (w/v) in the solution. Control groups received a salt mixture (NaCl/KCl) containing concentrations of sodium and potassium in water equivalent to those of the βOHB sodium/potassium group. Food intake, water consumption, and body weight of mice were recorded regularly.

### 4.6 Metabolic and body composition measurement

Whole‐body metabolic measurements were conducted using the OxyletPro System (Panlab). Mice were individually housed in the metabolic cage with free access to food and water. Light schedule, humidity, and temperature were the same as in the home cages. For indirect calorimetry measurements, O_2_ and CO_2_ levels were measured at 9‐minute intervals for a period of 180 s, with a controlled air supply at a flow rate of 300 ml/min. Activity levels were recorded by the transducer platform every 3 min. All data were analyzed using Metabolism software (Ver. 3.0, Panlab). Fat and lean mass of mice were measured by Time Domain NMR using Minispec LF‐50 TD‐NMR Body Composition Analyzer (Bruker) in the Taiwan Mouse Clinic.

### 4.7 Glucose, ATP/ADP, triglyceride, cholesterol, and βOHB measurements

Experimental mice were fasted for 10 h prior to testing. Basal blood glucose levels were measured at time 0 from tail vein with a glucometer (Bionime Rightest GM300), after which 1.5 g/kg glucose (Sigma) or 0.6 U/kg insulin (Sigma) was injected intraperitoneally (IP). Blood glucose levels were then measured at 15, 30, 60, 90, and 120 min after IP injection. Fly hemolymph samples were collected, and glucose levels were measured as described previously (Huang et al., 2015). Levels of βOHB in fly and mouse blood were measured using a ketone monitoring system (FreeStyle Optium, Abbott) as described previously (Teng et al., 2019). ATP, ADP, triglyceride, and cholesterol levels from whole flies or mouse liver tissues were measured using kits following the manufacturer's instructions (BioVision).

### 4.8 Golgi‐Cox impregnation and oil red O staining

Morphologic features of mouse hippocampus dentate gyrus (DG) granule cells were visualized using the FD Rapid GolgiStain™ kit (FD NeuroTechnologies). Dendritic morphology and spine density were analyzed with Neurolucida software (MBF, Bioscience), as described previously (Teng et al., 2019). For oil red O staining of liver, tissues were fixed in 4% paraformaldehyde (Sigma) and embedded in Cryo‐Gel embedding medium. 10μm sections were cut and stained with Oil Red O solution (Sigma) for 15 min. Stained sections were visualized using an Axio Imager M2 microscope, and photographs were taken by AxioCam ICc1 with AxioVision software (Zeiss).

### 4.9 mRNA quantification

Total RNA was isolated from liver tissue of each mouse using the NucleoSpin RNA Kit (Macherey‐Nagel), and cDNA was generated using oligo‐d(T)_15_ (Invitrogen) and SuperScript III reverse transcriptase (Invitrogen), as described previously (Lin et al., 2018). Quantitative PCR was carried out using a StepOnePlus Real‐Time PCR System (Applied Biosystems), SYBR Green Master Mix (Fermentas), and gene‐specific primers (Table S1).

### 4.10 Behavioral tests

All behavioral tests were performed in the dark phase. The open field and novel object recognition tests were performed as described previously (Teng et al., 2019). In the rotarod test, experimental mice were made to walk on a rotating rod to assess their motor coordination. Mice were first allowed to habituate on the static rod for 5 minutes, followed by 3 sessions of the training phase, with rod spinning at 5 rpm for 5 min. In the testing phase, the mice were tested under speeds of 10, 20, and 30 rpm for up to 5 min, and the latency to falling off the rod was recorded. The inter‐trial interval throughout the entire training and testing phases was set at 5 min.

The elevated plus maze test was conducted in a plus‐shaped apparatus, consisting of four arms and a central area, elevated above the floor at a height of 25 cm. The arms measure 40 cm long by 6 cm wide and comprise two oppositely positioned open arms with 1.5 cm high walls, and the two oppositely positioned closed arms with 25.5 cm high walls. Mice were allowed to explore freely in the maze for 5 min. The amount of time spent in the open and closed sets of arms was recorded.

The tail suspension test was conducted in a white plexiglass box measuring 40 cm in all dimensions. The experimental mouse was hung by its tail, using tape at the center of the top wall. Testing time was 6 min, and the time each mouse spent struggling was recorded. The forced swim test was carried out in a transparent cylinder, 30 cm high and 20 cm in diameter, filled with water to a height of 15 cm to prevent mice from reaching the floor. Mice were put into the water for 6 min, and struggling times were recorded during the last 4 min. For the buried food test, 24‐hour‐fasted mice were first allowed to habituate for 5 min in a testing cage with clean bedding. Following habituation, a 2 g food pellet was buried in one corner of the cage, and the mice were returned to the center of the cage. Latency or time taken by each mouse to find the hidden food was recorded.

The three‐chamber social test was performed in a rectangular box, divided into three compartments by two transparent walls that have an opening in the middle allowing the mice to freely access any chamber. Two containers were placed in the upper part of the two outer chambers for situating the stimulus mouse. The experimental mice were first allowed to habituate in the box for 30 min, with the two stimulus containers left empty. During the sociability testing phase, one new stimulus mouse (mouse A) was placed in the container in the left, and the subject mouse was then put back into the box for 10 min. Time spent interacting with mouse A and the empty container was recorded. Next, in the social novelty testing phase, an additional stimulus mouse (mouse B) was placed in the container on the right. The subject mouse was again allowed to interact with the two stimulus mice, and interacting times were recorded. Finally, the social memory testing phase was conducted 24 h later. The subject mouse was put back into the box and allowed to interact with a familiar stimulus (mouse A) and another novel stimulus mouse (mouse C) for 10 min, and the interacting times were recorded. The discrimination index was calculated by subtracting exploring time for the familiar stimulus from the exploring time for the novel stimulus, and then dividing by the total time spent exploring both stimuli.

### 4.11 Statistical analysis

Survival curves were analyzed using the Mantel‐Cox (log‐rank) test. All other data are expressed as mean ± SEM and were examined by Student's *t* test or one‐way ANOVA with Fisher's LSD post hoc test (StatPlus:mac). The statistical details of experiments are described in the figure legends, Tables S2, and S3.

## CONFLICT OF INTEREST

The authors declare no conflict of interest.

## AUTHOR CONTRIBUTIONS

CSL, YWW, SRY, YST, and ACL conducted the experiments. SZF and WSL contributed to the study discussion and manuscript editing. PYW and SZF designed and supervised the experiments. PYW wrote the manuscript. All authors read and approved the final manuscript.

## Supporting information

Fig S1–S8 (Please check the figure legends of Fig S3 and S6. Figures are placed on top of words. The color of words can be changed to black, some are in blue colors due to the previors revision)

Table S1

Table S2(The color of words can be changed to black, some are in blue colors due to the previors revision.)

Table S3 (The color of words can be changed to black, some are in blue colors due to the previors revision.)

## Data Availability

The data that support the findings of this study are available from the corresponding author upon reasonable request.
