# Supplementary material for: Dietary citrate supplementation enhances longevity, metabolic health, and memory performance through promoting ketogenesis
Source: Aging Cell. 2021 Oct 31;20(12):e13510. doi: 10.1111/acel.13510 (PMC8672782; doi:10.1111/acel.13510)
Supplement: Supplementary file 1 — Fig S1–S8 (Please check the figure legends of Fig S3 and S6. Figures are placed on top of words. The color of words can be changed to black, some are in blue colors due to the previors revision) [file ACEL-20-e13510-s004.docx]

**Figure S1. Citrate-induced lifespan extension in female flies is associated with AMPK and TOR pathways.** (A and B) Lifespans of female mutant flies with RU486-induced systemic inhibition of AMPK (A, *Tub-GS>UAS-AMPK RNAi*) and dTOR (B, *Tub-GS>UAS-dTOR^TED^*), treated with vehicle or 0.1% citrate. (D and E) Lifespans of female mutant flies with RU486-induced fat-body-specific inhibition of AMPK (D, *S106>UAS-AMPK RNAi*) and dTOR (E, *S106>UAS-dTOR^TED^*), treated with vehicle or 0.1% citrate. (C and F) Lifespans of female mutant flies with RU486-induced systemic (C, *Tub-GS>UAS-GFP*) and fat-body-specific (F, *S106>UAS-GFP*) overexpression of GFP, treated with vehicle or 0.1% citrate. Detailed statistical analyses for the lifespans are shown in Table S2.

**Figure S2. *Drosophila* citrate transporter *Indy* is required for citrate-induced lifespan extension.** Lifespans of male (A) and female (B) homozygous *Indy 206* mutant flies, treated with vehicle or 0.1% citrate. Detailed statistical analyses for the lifespans are shown in Table S2.

**Figure S3. Citrate-induced lifespan extension in uninduced mutant flies carrying different RU486-inducible UAS-Gal4 transgenes.** Lifespans of RU486-uninduced male (A, C, E, G, I, K, M and O) and female (B, D, F, H, J, L, N and P) mutant flies carrying *Tub-GS* (A, B, E, F, I, J, M and N) or *S106* (C, D, G, H, K, L, O and P) and *UAS-AMPK RNAi* (A-D), *UAS-dTOR^TED^* (E-H), *UAS-PGC-1α* *RNAi* (I-L) or *UAS-Hmgcl RNAi* (M-P) transgenes, treated with vehicle or 0.1% citrate. Detailed statistical analyses for the lifespans are shown in Table S2.

**Figure S4. AMPK overexpression in *Drosophila* fat body does not augment citrate-induced lifespan extension.** Lifespans of RU486-induced or uninduced male (A) and female (B) mutant flies carrying *S106>UAS-mCherry-AMPK*, treated with vehicle or 0.1% citrate. Detailed statistical analyses for the lifespans are shown in Table S2.

**Figure S5. Ketogenesis mediates citrate-induced lifespan extension in female flies.** (A and B) Lifespans of female mutant flies with RU486-induced systemic inhibition of PGC-1α (A, *Tub-GS>UAS-PGC-1α* *RNAi*) and Hmgcl (B, *Tub-GS>UAS-Hmgcl RNAi*), treated with vehicle or 0.1% citrate. (C and D) Lifespans of female mutant flies with RU486-induced fat-body-specific inhibition of PGC-1α (C, *S106>UAS-PGC-1α* *RNAi*) and Hmgcl (D, *S106>UAS-Hmgcl RNAi*), treated with vehicle or 0.1% citrate. (E and F) Citrate treatment for 10 days increases βOHB levels in female genetic control flies (*Tub-GS/S106>UAS-GFP*), but not in female mutant flies with systemic and fat-body-specific overexpression of AMPK RNAi (*Tub-GS/S106>UAS-AMPK RNAi*), dTOR^TED^ (*Tub-GS/S106>UAS-dTOR^TED^*), PGC-1α RNAi (*Tub-GS/S106>UAS-PGC-1α RNAi*) and HMGL RNAi (*Tub-GS/S106>UAS-Hmgcl RNAi*). (G) Dietary βOHB supplementation for 10 days dose-dependently increases βOHB in female flies. (H) Lifespans of female *w^1118^* flies treated with different concentrations of βOHB. Detailed statistical analyses for the lifespans are shown in Table S2 and S3. The other data are expressed as mean ± SEM (n=9-10 samples). *P<0.05, **P<0.01, ***P<0.001 compared to the control group by Student’s t test or one-way ANOVA with Fisher's LSD post hoc test.

**Figure S6. Whole body metabolic analyses in mice treated with vehicle, citrate and βOHB.** O_2_ consumption (A, D, and G), CO_2_ production (B, E, and H) and respiratory quotient (C, F, and I) of mice receiving different concentrations of citrate (A-C) and βOHB (D-I) treatment. Data are expressed as mean ± SEM (n=4-8 mice). Not significant (n.s.), **P<0.01 compared to the control group by Student’s t test or one-way ANOVA with Fisher's LSD post hoc test.

**Figure S7. Citrate treatment does not affect inguinal white adipose tissue.** Representative micrographs (A) and average weight (B) of inguinal white adipose tissue from mice treated with different concentrations of citrate are shown. Data are expressed as mean ± SEM (n=10 mice per group). Not significant (n.s.) compared to the control group by one-way ANOVA with Fisher's LSD post hoc test.

**Figure S8. βOHB administration affects mouse behavior.** Mice fed on a high-fat diet were treated with different concentrations of vehicle and βOHB from 10 weeks of age. Behavioral tests were carried out from 16-24 weeks of age. (A) Mice were subjected to the open field test (A), rotarod test (B and C), elevated plus maze test (D), tail suspension test (E), forced swim test (F), buried food test (G), 3-chamber social test (H and I). Data are expressed as mean ± SEM (n=8-10 mice per group). Not significant (n.s.), *P<0.05, **P<0.01 compared to the control group by Student’s t test.
