# Supplementary material for: Dietary citrate supplementation enhances longevity, metabolic health, and memory performance through promoting ketogenesis
Source: Aging Cell. 2021 Oct 31;20(12):e13510. doi: 10.1111/acel.13510 (PMC8672782; doi:10.1111/acel.13510)
Supplement: Supplementary file 2 — Table S1 [file ACEL-20-e13510-s003.docx]

Table S1. Primer sequences used for qPCR analysis.

| Gene name | Primer sequences (5’-3’) |
| --- | --- |
| ATP citrate lyase | F: AATGGCCGTCATGTGAGTTT  R: GTGGCCCCAACTATCAAGAG |
| Acetyl-CoA carboxylase | F: CACCAACGCTAAAGTGGCG  R: CCGAGAGGATGAGTTTCTGC |
| Fatty acid synthase | F: GGCCCCTCTGTTAATTGGCT  R: CGCTTGTTGGTGGACACTTG |
| HMG-CoA synthetase 1 | F: TGATCCCCTTTGGTGGCTGA  R: AGGGCAACGATTCCCACATC |
| HMG-CoA reductase | F: ATCCTGACGATAACGCGGTG  R: AAGAGGCCAGCAATACCCAG |
| Mevalonate diphosphate decarboxylase | F: CTGCACCAGGACCAGCTAAA  R: CTGAGGCTGAGGGGTAGAGT |
| HMG-CoA synthetase 2 | F: AGAGAGCGATGCAGGAAACTT  R: AAGGATGCCCACATCTTTTGG |
| HMG-CoA lyase | F: ACTACCCAGTCCTGACTCCAA  R: TAGAGCAGTTCGCGTTCTTCC |
| β-hydroxybutyrate dehydrogenase 1 | F: GGTGGAACCTGGCAACTTCAT  R: GGTCATCCCACATCTTCTTGG |
| β-actin | F: GGCACCACACTTCTACAATG  R: GGGGTGTTGAAGGTCTCAAAC |
