# Supplementary material for: Dietary citrate supplementation enhances longevity, metabolic health, and memory performance through promoting ketogenesis
Source: Aging Cell. 2021 Oct 31;20(12):e13510. doi: 10.1111/acel.13510 (PMC8672782; doi:10.1111/acel.13510)
Supplement: Supplementary file 3 — Table S2(The color of words can be changed to black, some are in blue colors due to the previors revision.) [file ACEL-20-e13510-s002.docx]

Table S2. Lifespans of *Drosophila* treated with different concentrations of citrate.

| Genotype | Gender | Citrate (%) | n | Mean lifespan (d) | Extension (%) | P value |
| --- | --- | --- | --- | --- | --- | --- |
| *w1118* | Male | 0 | 497 | 47.27 |  |  |
|  |  | 0.05 | 185 | 51.88 | 9.75 | *** |
|  |  | 0.1 | 509 | 55.79 | 18.02 | *** |
|  |  | 1 | 542 | 54.77 | 15.87 | *** |
|  | Female | 0 | 517 | 47.82 |  |  |
|  |  | 0.05 | 204 | 55.50 | 16.06 | *** |
|  |  | 0.1 | 559 | 56.14 | 17.40 | *** |
|  |  | 1 | 558 | 56.03 | 17.17 | *** |
| *Tub-GS>UAS-GFP* | Male | 0 | 428 | 23.94 |  |  |
| induced |  | 0.1 | 424 | 29.04 | 21.30 | *** |
|  | Female | 0 | 434 | 29.53 |  |  |
|  |  | 0.1 | 432 | 34.72 | 17.58 | *** |
| *S106-GS>UAS-GFP* | Male | 0 | 439 | 43.87 |  |  |
| induced |  | 0.1 | 433 | 48.51 | 10.58 | *** |
|  | Female | 0 | 426 | 32.69 |  |  |
|  |  | 0.1 | 434 | 39.91 | 22.09 | *** |
| *S106-GS>UAS-mCherry-AMPK* | Male | 0 | 219 | 42.33 |  |  |
| induced |  | 0.1 | 210 | 41.58 | -1.77 | ns. |
|  | Female | 0 | 224 | 41.35 |  |  |
|  |  | 0.1 | 215 | 40.42 | -2.25 | ns. |
| *S106-GS>UAS-mCherry-AMPK* | Male | 0 | 217 | 36.56 |  |  |
| uninduced |  | 0.1 | 219 | 41.58 | 13.73 | *** |
|  | Female | 0 | 211 | 34.46 |  |  |
|  |  | 0.1 | 227 | 39.42 | 14.39 | *** |
| *Tub-GS>UAS-AMPK RNAi* | Male | 0 | 418 | 25.87 |  |  |
| induced |  | 0.1 | 439 | 25.15 | -2.78 | *** |
|  | Female | 0 | 435 | 25.67 |  |  |
|  |  | 0.1 | 441 | 24.79 | -3.43 | ns. |
| *Tub-GS>UAS-AMPK RNAi* | Male | 0 | 222 | 36.88 |  |  |
| uninduced |  | 0.1 | 234 | 40.29 | 9.25 | *** |
|  | Female | 0 | 223 | 41.50 |  |  |
|  |  | 0.1 | 252 | 47.32 | 14.02 | *** |
| *S106-GS>UAS-AMPK RNAi* | Male | 0 | 424 | 29.95 |  |  |
| induced |  | 0.1 | 417 | 29.14 | -2.70 | ns. |
|  | Female | 0 | 425 | 25.28 |  |  |
|  |  | 0.1 | 434 | 24.41 | -3.44 | ** |
| *S106-GS>UAS-AMPK RNAi* | Male | 0 | 217 | 25.17 |  |  |
| uninduced |  | 0.1 | 223 | 29.74 | 18.16 | *** |
|  | Female | 0 | 219 | 22.92 |  |  |
|  |  | 0.1 | 208 | 27.68 | 20.77 | *** |
| *Tub-GS>UAS-dTor^TED^* | Male | 0 | 414 | 30.71 |  |  |
| induced |  | 0.1 | 423 | 29.48 | -4.01 | ** |
|  | Female | 0 | 428 | 29.38 |  |  |
|  |  | 0.1 | 419 | 28.56 | -2.79 | *** |
| *Tub-GS>UAS-dTor^TED^* | Male | 0 | 206 | 33.82 |  |  |
| uninduced |  | 0.1 | 219 | 38.23 | 13.04 | *** |
|  | Female | 0 | 270 | 35.45 |  |  |
|  |  | 0.1 | 227 | 39.23 | 10.66 | *** |
| *S106-GS*>*UAS-dTor^TED^* | Male | 0 | 439 | 51.37 |  |  |
| induced |  | 0.1 | 433 | 51.39 | 0.04 | ns. |
|  | Female | 0 | 447 | 44.59 |  |  |
|  |  | 0.1 | 438 | 45.78 | 2.67 | ns. |
| *S106-GS*>*UAS-dTor^TED^* | Male | 0 | 246 | 43.04 |  |  |
| uninduced |  | 0.1 | 209 | 51.53 | 19.73 | *** |
|  | Female | 0 | 235 | 44.36 |  |  |
|  |  | 0.1 | 212 | 48.71 | 9.81 | *** |
| *Tub-GS*>*UAS PGC-1α* *RNAi* | Male | 0 | 438 | 24.12 |  |  |
| induced |  | 0.1 | 437 | 24.45 | 1.37 | ns. |
|  | Female | 0 | 443 | 29.12 |  |  |
|  |  | 0.1 | 430 | 29.33 | 0.72 | ns. |
| *Tub-GS*>*UAS PGC-1α* *RNAi* | Male | 0 | 174 | 28.92 |  |  |
| uninduced |  | 0.1 | 210 | 34.95 | 20.85 | *** |
|  | Female | 0 | 217 | 32.30 |  |  |
|  |  | 0.1 | 201 | 41.12 | 27.31 | *** |
| *S106-GS*>*UAS PGC-1α RNAi* | Male | 0 | 430 | 39.36 |  |  |
| induced |  | 0.1 | 434 | 37.33 | -5.16 | * |
|  | Female | 0 | 433 | 32.41 |  |  |
|  |  | 0.1 | 434 | 30.71 | -5.25 | * |
| *S106-GS*>*UAS PGC-1α RNAi* | Male | 0 | 348 | 35.00 |  |  |
| uninduced |  | 0.1 | 420 | 44.16 | 26.17 | *** |
|  | Female | 0 | 317 | 25.67 |  |  |
|  |  | 0.1 | 333 | 35.59 | 38.64 | *** |
| *Tub-GS*>*UAS-Hmgcl RNAi* | Male | 0 | 339 | 31.28 |  |  |
| induced |  | 0.1 | 344 | 29.56 | -5.50 | *** |
|  | Female | 0 | 330 | 28.82 |  |  |
|  |  | 0.1 | 336 | 27.19 | -5.66 | *** |
| *Tub-GS*>*UAS-Hmgcl RNAi* | Male | 0 | 209 | 34.16 |  |  |
| uninduced |  | 0.1 | 179 | 41.21 | 20.64 | *** |
|  | Female | 0 | 215 | 31.82 |  |  |
|  |  | 0.1 | 235 | 38.59 | 21.28 | *** |
| *S106-GS*>*UAS-Hmgcl RNAi* | Male | 0 | 406 | 40.50 |  |  |
| induced |  | 0.1 | 386 | 37.89 | -6.44 | *** |
|  | Female | 0 | 417 | 30.59 |  |  |
|  |  | 0.1 | 396 | 29.73 | -2.81 | ns. |
| *S106-GS*>*UAS-Hmgcl RNAi* | Male | 0 | 223 | 47.89 |  |  |
| uninduced |  | 0.1 | 219 | 53.22 | 11.13 | *** |
|  | Female | 0 | 247 | 36.68 |  |  |
|  |  | 0.1 | 209 | 40.81 | 11.26 | *** |
| *Indy 206* | Male | 0 | 223 | 52.33 |  |  |
|  |  | 0.1 | 224 | 53.14 | 1.55 | ns. |
|  | Female | 0 | 203 | 54.31 |  |  |
|  |  | 0.1 | 209 | 54.05 | -0.48 | ns. |

Survival curves were analyzed using the Mantel-Cox (log-rank) test. Not significant (ns.), *P < 0.05, **P < 0.01, ***P < 0.001.
