# Supplementary material for: Dietary citrate supplementation enhances longevity, metabolic health, and memory performance through promoting ketogenesis
Source: Aging Cell. 2021 Oct 31;20(12):e13510. doi: 10.1111/acel.13510 (PMC8672782; doi:10.1111/acel.13510)
Supplement: Supplementary file 4 — Table S3 (The color of words can be changed to black, some are in blue colors due to the previors revision.) [file ACEL-20-e13510-s001.docx]

Table S3. Lifespans of *Drosophila* treated with different concentrations βOHB.

| Genotype | Gender | βOHB (mM) | n | Mean lifespan (d) | Extension (%) | P value |
| --- | --- | --- | --- | --- | --- | --- |
| *w1118* | Male | 0 | 433 | 37.93 |  |  |
|  |  | 20 | 417 | 41.63 | 9.75 | *** |
|  |  | 50 | 434 | 40.89 | 7.80 | *** |
|  | Female | 0 | 448 | 44.22 |  |  |
|  |  | 20 | 422 | 49.09 | 11.01 | *** |
|  |  | 50 | 421 | 46.29 | 4.68 | *** |

Survival curves were analyzed using the Mantel-Cox (log-rank) test. ***P < 0.001.
